# The effectiveness of a monetary reimbursement model for weight reduction via a smartphone application: a preliminary retrospective study

**DOI:** 10.1038/s41598-020-72908-5

**Published:** 2020-09-24

**Authors:** Jungeun Lee, Sujin Bae, Dohyung Park, Youngin Kim, Jisun Park

**Affiliations:** 1 Noom Inc, Seoul, 07325 Korea; 2 Ingenium College of Liberal Arts, KwangWoon University, Seoul, 01897 Korea; 3 Office of Research, Chung-Ang University, Seoul, 06974 Korea; 4 Department of Biomedical Systems Information, Yonsei University College of Medicine, Seoul, 03722 Korea; 5 Department of Social Work and Counseling, Catholic University of Pusan, Pusan, 46252 Korea

**Keywords:** Psychology, Health care

## Abstract

Weight loss for obese populations has been a challenging subject. There are numerous mobile applications to address weight loss, but the low retention rate is a barrier for the intervention. This is a retrospective study, aiming to investigate the effectiveness of financial incentives to achieve weight loss via a monetary reimbursement model on a smartphone application. Participants voluntarily purchased a 16-week mobile weight loss application program, and those who logged food intake three times a day received monetary reimbursement up to the full amount they initially paid. We analyzed health-related information and logged in-app activities from participants (N = 2,803) including age, sex, weight, food intake, and physical activity on their mobile healthcare application called Noom from January 2017 to April 2019. Analysis of covariance (ANCOVA) was used to compare differences between groups who succeeded and failed at food logging, controlling for baseline BMI. The ANCOVA found that participants who completed the food logging successfully for 16 weeks (N = 1,565) lost significantly more weight than those who failed food logging (N = 1,238, F = 56.0, *p* < 0.001). In addition, participants who were able to log their food intake successfully exercised more (F = 41.5, *p* < 0.001), read more in-app articles (F = 120.7, *p* < 0.001), and consumed more quantity of healthy foods (F = 12.8, *p* < 0.001). Monetary reimbursement is an effective tool for weight reduction by encouraging participants to monitor their health-related behaviors regularly.

## Introduction

Obesity is a major cause of cardiovascular disease, diabetes mellitus, high blood pressure, sleep disorders, musculoskeletal disease, arthritis, and several forms of cancer^1,2^. In 2016, 13% of adults worldwide had obesity and 39% of adults were overweight, which tripled since 1975^3^. In South Korea, the rate of obesity has been rapidly increasing. With proposed classification of weight by BMI in adult Asians from the WHO^4^ (obesity of BMI > 25), 34.1% of Korean adults (41.65% for male, 25.6% for female) were obese in 2017^5^.

Previous studies have shown that an unhealthy diet and lack of physical activity are the major causes of obesity^6^. Even though people understand the importance of a balanced diet and regular exercise, it is still challenging for them to engage in such healthy lifestyle regimes. It takes time, effort, and cost to prepare healthy meals composed of seasonal fruits and vegetables. Exercise is another hurdle to engage in to build a good lifestyle due to time constraints and discomfort with exercising^7^. In the United States, only 5% exercise enough to maintain physical health leaving 95% of the population lacking adequate physical activities^8^.

Monetary incentive, in which individuals are given incentives when engaging in health-related behaviors, has been receiving attention from researchers. Financial incentives are known to assist with treatment compliance^9^, dietary behavior^10^, and physical activity^11^. Mantzari et al. conducted a meta-analysis on 34 studies that used monetary incentives for health-related behaviors including weight-loss, and found financial incentive helped to motivate participants engage in healthy behaviors and maintain them^12^. Specifically, financial incentives improved weight-loss up to 12 months from the start of intervention. Thus, external motivational sources, such as monetary incentive, can be considered as an effective tool for weight loss.

Mobile health (e.g., smartphones, tablets, activity monitors) is an emerging mode of behavioral intervention delivery. Especially, numerous mobile applications have been developed as alternative tools to help lose weight since they offer a convenient platform to monitor and track food intake, weight, and progress. Based on recently published studies, the effectiveness of mobile healthcare application for weight reduction has been proven. For example, Rhee et al. reported that 77.9% of 35,921 application users reported decreased body weight while using the application, and 22.7% of them reported > 10% weight reduction (with a median application usage duration of 272 days)^13^. Michaelides et al. applied a diabetes prevention program via a smartphone application to 43 participants who were diagnosed with pre-diabetes due to overweight or obesity, and the program completers (N = 34) lost 7.5% (7.1 kg) of their initial weight^14^. A study by Kim et al.^15^ found that their smartphone application helped South Korean middle-aged females with chronic disease record their food intake and physical activities, and their weight, body fat, BMI, WHR, and LDL significantly decreased after the 12-week exercise program.

Although there are many benefits of using weight loss interventions on mobile applications, maintaining engagement of users over time has been a challenging topic^16^. Users who did not experience satisfactory weight reduction in the early phase of application usage are highly likely to terminate continued involvement during the initial stage of the program^17,18^. To address high attrition rates of mobile applications, we added a factor of the deposit contract in this study as a form of reimbursement (i.e., monetary incentive) to achieve higher engagement in the weight loss program. Since food logging was proven as one of the key behaviors for weight loss^13,14^, we set that behavior as a goal for study participants to achieve. In this study, we hypothesized that participants who successfully log their food intake regularly (three times a day for 16 weeks) while anticipating reimbursement would lose more weight compared to those who fail regular food logging. In addition, we examined if other health-related behaviors on the application (*e.g.*, average working out hours, total hours reading health-related information, composition of food consumption) were different between the two groups.

## Results

### Recruitment and enrollment

A total of 3,046 participants enrolled from January 2017 to April 2019. Among them, we limited the participants into those who logged their weight at week 0 (starting point), week 8 and week 16 (end point) to examine weight reduction. In addition, we excluded participants who exceeded three standard deviations from the mean regarding values of age, baseline BMI, weight change in total, physical activity in min per week (median). This left 2,803 participants for data analysis. The mean (SD) age of the participants was 33.6 (7.9); 87% was women; the mean starting weight was 65.2 kg (10.2). Retrospectively, there were statistically significant differences between the two groups regarding height, weight, and baseline BMI (Table 1).

**Table 1:** Baseline characteristics of the study participants.

	Mission success (N = 1,565)	Mission failure (N = 1,238)	t (*x*^*2*^)	*p*
Age (years)	33.4 (8.0)	33.9 (7.9)	−1.90	0.06
Height (cm)	163.5 (6.9)	164.1 (6.7)	−2.04	0.04
Weight (kg)	64.6 (10.1)	66.0 (10.4)	−3.48	0.001
Baseline BMI (kg/m^2^)	24.1 (2.7)	24.4 (2.8)	−3.37	0.001
Underweight (BMI < 18.5)	2	6	(14.56)	0.002
Normal (18.5–25)	1068	769		
Overweight (25–30)	447	410		
Obesity (> 30)	48	53		

*BMI*  body mass index.

### Overall weight outcome

Among all participants, 42.1% of them lost more than 3% of their initial weight. In addition, more than 1/5 of the participants (22.2%) lost more than 5% of their weight compared to their initial weight.

### Difference between the mission success and mission failure groups on weight

**Figure 1:**
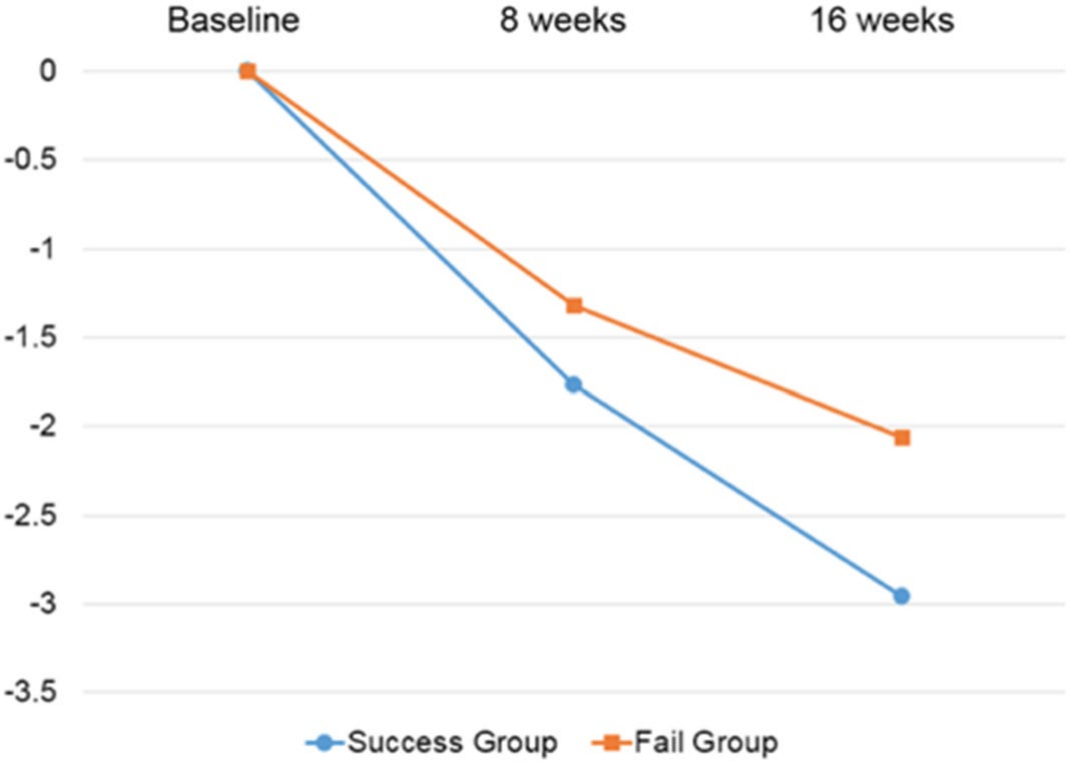
Percent weight change from entry over time.

We divided the participants into two groups based on mission outcomes. The participants who were successful with their missions (i.e., completed food logging three times a day throughout the 16 weeks without missing any logging) were defined as the mission success group (55.8%) and participants who failed at least one mission was defined as mission failure group (44.2%). Weight change was significantly different between the two groups from baseline to 16 weeks (F = 56.0, *p* < 0.001). Specifically, participants in the mission success group lost 2.96% of their baseline weight while those in mission failure group lost 2.06% of their baseline weight (see Table 2). We also looked at weight difference at week 8 from participants’ baseline weight; the mission success group’s participants lost more weight than those in mission failure group (1.77% vs. 1.32%, t = -5.74, *p* < 0.001). Percent weight change between the two groups over time can be seen in Fig. 1.

**Table 2:** Difference of weight reduction between the mission success and failure groups.

	Mission success	Mission failure	F (t)	*p*	*η*
Weight change in total (%) (baseline to week 16)	−2.96 (3.42)	−2.06 (3.23)	56.0	< 0.001	0.020
Normal weight group	−2.85 (3.45)	−1.87 (3.10)	42.0	< 0.001	0.022
Excess weight group	−3.23 (3.32)	−2.39 (3.40)	15.2	< 0.001	0.016
Baseline weight (kg)	64.6 (10.1)	66.0 (10.4)	(−3.48)	0.001	–
Normal weight group	60.0 (5.79)	60.5 (5.57)	(−2.17)	0.03	–
Excess weight group	74.8 (9.95)	75.3 (9.84)	(−0.73)	0.47	–
Week 8 weight change (%)	−1.77 (2.10)	−1.32 (1.98)	(−5.74)	< 0.001	–
Normal weight group	−1.75 (2.08)	−1.23 (1.91)	(−5.49)	< 0.001	–
Excess weight group	−1.83 (2.13)	−1.47 (2.09)	(−2.63)	0.009	–

16-week engagement-in-app activities					
Physical activity in min per week (median)	151 (100)	128 (88)	41.5	< 0.001	0.015
Articles read	277 (130)	224 (120)	120.7	< 0.001	0.041
Meals logged (meals per week)	26.87 (3.70)	22.56 (5.79)	535.1	< 0.001	0.160
Energy intakes (kcal, average for 16-week)	1209 (271)	1183 (285)	9.4	0.002	0.003
Carbohydrates (g, average for 16-week)	159.1 (56)	153.4 (46)	10.0	0.002	0.004
Protein (g, average for 16-week)	57.3 (20)	54.8 (17)	16.8	< 0.001	0.006
Fat (g, average for 16-week)	41.1 (13)	39.7 (12)	11.2	0.001	0.004
Food intake proportion Green color average (total)	16.8 (7.6)	15.8 (7.4)	12.8	< 0.001	0.005
Yellow color average (total)	47.8 (6.8)	48.1 (7.3)	0.6	0.45	–
Red color average (total)	33.3 (8.6)	34.4 (8.7)	12.0	0.001	0.004

The F statistics came from ANCOVA controlling for baseline BMI.

In addition, we divided mission success and failure groups into normal weight and excess weight groups separately to see if different conditions of starting weight are related to the weight change outcome. As a result, the excess weight group lost 1.83% of their initial weight at week 8 and 3.23% of their starting weight at week 16, while the normal weight group lost 1.75% of their initial weight at week 8 and 2.85% of their starting weight at week 16.

### Secondary outcomes

**Figure 2:**
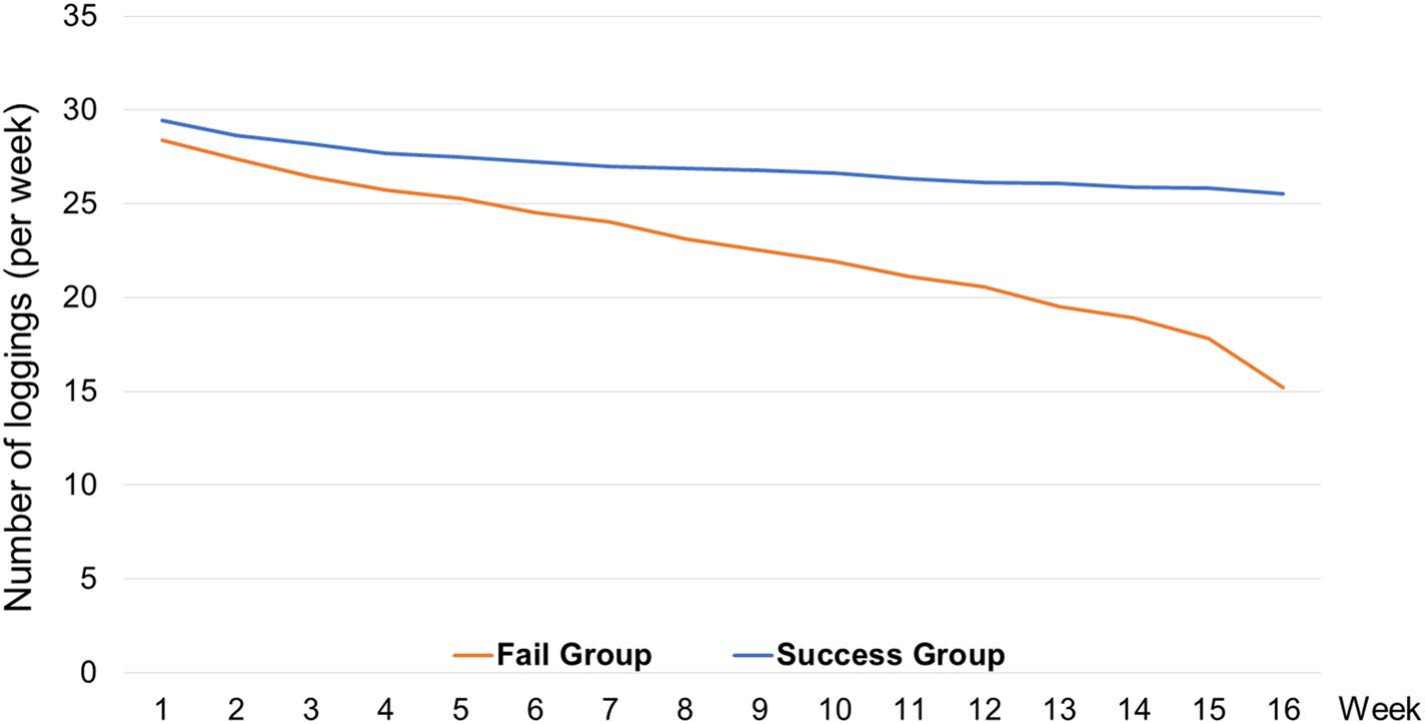
Number of meal loggings per week of mission failure and success group.

We examined participants’ other health-related behaviors while using the Noom application such as their average length of weekly physical activity, the total number of health-related articles read, number of total food logging, and food intake composition. The mission success group spent significantly more time working out than the failure group (151 min vs. 128 min per week, F = 41.5, *p* < 0.001), and total articles read was significantly higher for the mission success group compared to the mission failure group (277 articles vs. 224 articles, F = 120.7, *p* < 0.001). Additionally, the total average food loggings per week in the mission success group was significantly higher than the other group (26.97 vs. 22.65, F = 4.52, *p* < 0.001). As seen in Fig. 2, participants in the mission success group were able to continue logging their food intake consistently throughout 16 weeks. We also analyzed total 16-week average energy intake and nutrients (*i.e.*, carbohydrates, protein, fat) for each group. As a result, the mission success group consumed higher energy intake (1,209 kcal vs. 1,184 kcal, F = 9.4, *p* = 0.002), carbohydrates (159.1 g vs. 153.4 g, F = 10.0, *p* = 0.002), protein (57.3 g vs. 54.8 g, F = 16.8, *p* < 0.001) and fat (41.1 g vs. 39.7 g, F = 11.2, *p* = 0.001) compared to the mission failure group. Additionally, proportion of food intake for each group with color labels of green, yellow, and red were analyzed. In the Noom application, foods are classified into red, green and yellow based on caloric density. Specifically, food with high density are labeled with the color red (kcal of food / gram of food ≥ 2.5) and low density foods are labeled with the color green (kcal of food / gram of food ≤ 1.0), leaving the middle as yellow (1.0 < kcal of food / gram of food < 2.5). In this classification system, foods considered as healthy (i.e., seasonal fruits and vegetables) have low density and are labeled as green. Users are encouraged to consume more green color foods on a daily basis while red color foods are suggested to be consumed with caution. Mission success group consumed green color food in greater quantities (16.8% vs. 15.8%, F = 12.8, *p* < 0.001) and red color food lower (33.3% vs. 34.4%, F = 12.0, *p* = 0.001) than the mission failure group.

## Discussion

In this study, we examined the effectiveness of a monetary incentive with a deposit system for Noom application users (N = 2,803) to lose weight. As a result, 55.8% of them logged their food consumption three times a day for 16 weeks, and they lost significantly more weight than those who failed food logging during the same period (2.96% vs. 2.06% of initial weight loss). In addition, the former group spent significantly more time working out, read significantly more health-related articles and consumed healthy foods more than the latter group.

The rate of weight loss from this study was smaller than the previous studies that used the same mobile application (i.e., Noom). For example, Rhee et al. analyzed global participants’ data (N = 35,381) and their initial average weight was 80.3 kg (BMI = 28.5); 22.7% of them lost more than 10% of baseline weight^13^. In addition, Michaelides et al.^14^ found 7.5% of weight loss from 24-week diabetes prevention program from American overweight/obese adult participants (N = 43, initial weight as of 96.61 kg, BMI = 35.5). We assumed that participants of this study are non-clinical Korean populations whose mean starting weights were much lower than American or global data (average baseline weight as of 68.8 kg, BMI = 24.4), and it can affect the total percentage of weight loss.

To our knowledge, this is the first study that evaluated the effectiveness of a monetary incentive for a mobile weight loss program adapting the deposit contract model. Prior studies examining the efficacy of using incentives to promote weight loss were conducted only in face-to-face interventions. Running an in-person reimbursement model usually requires human resources in terms of assigning tasks, keeping track of behaviors, and implementing incentive. The smartphone application environment can offer easier and efficient ways to communicate with participants and operate the reimbursement model. Thus, this study adds value to the literature by examining the efficiency and effectiveness of the reimbursement model on the mobile application platform for healthy lifestyle intervention.

Another meaningful result of this study is that more than half of the participants completed the 16-week program with active in-app participation (i.e., food logging, weight logging, physical activity, and article reading). Considering the known high dropout rate in the early phase of using mobile applications for weight loss in general, this study demonstrates the potential utility and effectiveness of using a deposit contract system to increase in-app participation engagement on a mobile platform. Previous studies confirmed that weight loss could be enhanced by using deposit contracts, whereby participants paid monetary deposits and received all or a portion of the money back depending on the achieved results^10–12^. From a theoretical perspective, deposit contracts created a system of negative reinforcement, whereby a target behavior (such as in-app participation) is reinforced by the removal of a negative/aversive stimulus (i.e., removal of the loss of deposited money). In this study, we set the target behavior as food logging, not weight loss, and continuous food logging was assumed to make participants cognizant of their meals composition and total calories, thereby naturally affecting their subsequent meal decisions and exercise planning in a positive way and contributing to eventual weight lose.

Behavioral economics theory also supports the usage of monetary reimbursement for lifestyle intervention. Behavioral economics, a classification of economic theory complemented with insights of psychology, acknowledge that human beings form judgments in a biased way acting in favor of one’s immediate self-interest while sacrificing long-term well-being (e.g., health and better appearance)^19^. This explains why instant positive experiences are perceived as more attractive than those in the future^20^. In this study, we designed instant gratification as monetary incentive to be given to participants based on their self-monitoring behaviors (i.e., food logging), and this motivates them to continue engaging in target behaviors continuously. Thus, satisfactory experiences early in the program (i.e., monetary incentive) leads participants to continue using the program, followed by other healthy behaviors (e.g., working out, reading health-related articles, cautious food choices).

The limitations of this study should be noted. First of all, we do not know if the effects of the reimbursement program lasted after the 16 weeks of the program. Even though incentives provided the initial motivation for a healthy lifestyle over the 16-week intervention and participants would have experienced the positive aspects of a healthier lifestyle, maintenance of weight loss has been a challenging subject^21,22^. Future studies that evaluate the continuation of positive behavioral changes and post-program weight loss (or maintenance) without extrinsic incentives are needed. Secondly, this is a retrospective study using the collected data from application users, and we solely depended on users’ food, exercise, and weight loggings, which limits the accuracy of self-reports and generalizability. Further studies can utilize Bluetooth technology to increase the accuracy of collected information, especially weight reports, to replicate the result of this study. Thirdly, participants who failed at weight loggings (i.e., 8 week and 16 weeks) were not included in the analysis. Thus, we do not have information of the missing participants’ data of weight and health related activities. The last limitation is that there were statistically significant differences between the two groups in terms of baseline height, baseline weight, and BMI. From additional analysis by dividing initial normal weight and excess weight sub-groups, we found that only normal weight group participants’ starting weights were statistically significantly different between mission success and failure groups, leaving excess weight groups as homogeneous.

In conclusion, we demonstrated preliminary utility of a smartphone application for weight reduction in the form of monetary reimbursement from a large number of participants. Participants in this study voluntarily purchased the application to lose weight, which is consistent with individuals who use smartphone applications for healthcare, making this study generalizable to populations of weight loss application users. In future studies, evaluation of specific behavioral changes (e.g., specific change of portion/contents of meal, change of workout length/frequency/contents), continued engagement of new behaviors after the intervention, post-program weight loss without monetary reimbursement, and utility for clinical populations, will be necessary.

## Methods

### Mobile intervention

In this study, we used the Noom application as a mobile platform. The Noom application has been on the Google Playstore since 2012 and it has been ranked as one of the top weight reduction applications with 10 million downloads worldwide^23,24^. Chen et al. evaluated top 200-rated Health and Fitness category applications for weight management from Google Play and iTunes App Store in Australia in 2014 (N = 800) and reported that Noom had the highest score of accountability, information accuracy related to weight management, technology-enhanced features, usability and incorporations of behavior change techniques^25^. In addition, previous studies which used Noom data consistently reported that Noom is effective for weight loss^13,14^.

When starting Noom application program, users are asked to set their body weight goal and enter the current weight. There are also functions of recording meal, exercise, and weight as well as in-app human coaching. Human coaches interact with participants via in-app messages a couple of times per week to give feedback on food intake/nutrition and workout activities. In-app alarm encourages users to log their food intake, and in-app articles containing health-related information on a daily basis are additionally offered. In-app articles contain how to make smart food choices, introductions of several workout plans, and stress management strategies. Weight changes, nutritional summaries of one's diet, and recipes are other functions users can utilize.

### Study protocol

This is a retrospective study and based on data of participants who purchased and installed Noom application from January 2017 to April 2019. The analysts of this study (S. Bae) was provided with all relevant data by Noom after de-identifying the entire dataset. Users of application voluntarily logged in their health-related information including food intake, exercise and weight input.

Users paid a 3-month program fee ($120) when downloading the application. They received monetary incentives when logging food intake three times a day (referred to as missions) at week 1 ($3), week 2 ($6), week 4 ($10), week 8 ($15), and week 16 ($86). As long as they recorded their food consumption, financial incentive was given, regardless of weight loss. This was decided based on Rhee and his colleagues’ study (2016) which found that self-monitoring is a crucial factor of behavioral change, using the same mobile application environment^13^. We chose meal logging as a continuous self-monitoring behavior. In addition, reimbursement is given periodically five times through the course of the 16-week intervention, instead of one final amount at the end of study based on previous studies reporting that frequent rewards are more effective approach to provide monetary incentives^26^.

### Statistical analysis

We compared the differences in the baseline characteristics between the mission success and mission failure group using a t-test. We assessed the effectiveness of monetary incentives for weight loss using ANCOVA (analysis of covariance) between the two groups. The values of the baseline BMI were entered as a covariate. We also compared the differences of the secondary outcomes by ANCOVA controlling for baseline BMI.

### Ethics statement

This study was implemented according to the guidelines in the Declaration of Helsinki, the privacy of Noom Inc., and approved by the Pusan Catholic University Institutional Review Board (CUPIRB-2019–01-007), which confirmed the absence of risk for the de-identified personal information disclosure. The informed consent from the study participants were waived because of the retrospective design of the study.

## Data Availability

The datasets generated analyzed during the current study are available from the corresponding author on reasonable request.
